# Research Progress on the Mechanism for Improving Glucose and Lipid Metabolism Disorders Using Phenolic Acid Components from Medicinal and Edible Homologous Plants

**DOI:** 10.3390/molecules29204790

**Published:** 2024-10-10

**Authors:** Miao Sun, Zhimin Zhang, Jingchen Xie, Jiahui Yu, Suhui Xiong, Feng Xiang, Xinyi Ma, Chen Yang, Limei Lin

**Affiliations:** Key Laboratory for Quality Evaluation of Bulk Herbs of Human Province, School of Pharmacy, Hunan University of Chinese Medicine, Changsha 410208, China; 20223726@stu.hnucm.edu.cn (M.S.); cslgdxzzm@163.com (Z.Z.); xiejingchen2022@163.com (J.X.); 20223725@stu.hnucm.edu.cn (J.Y.); 20222058@stu.hnucm.edu.cn (S.X.); 20232050@stu.hnucm.edu.cn (F.X.); 20233765@stu.hnucm.edu.cn (X.M.); 20233766@stu.hnucm.edu.cn (C.Y.)

**Keywords:** homology of medicine and food, plant phenolic acids, glycolipid metabolism

## Abstract

Glucose and lipid metabolism disorders are the core pathological mechanism of a variety of metabolic diseases, and the incidence of related diseases is increasing year by year, which seriously threatens human life and health. Traditional Chinese medicine with medicinal and edible properties refers to Chinese medicinal resources that have both medicinal and edible characteristics. Due to its safety and its health-promoting and medicinal functions, traditional Chinese medicine has received increasing attention in the development of functional health foods. Phenolic acids are important secondary metabolites that are ubiquitous in medicinal and edible homologous plants, and the regulation of glycolipid metabolism is an important activity and plays a key role in many diseases. In this paper, we focus on the alleviation of glycolipid disorders using MEHH phenolic acids, which regulate glucose metabolism and lipid metabolism, improve insulin resistance, inhibit inflammatory responses, alleviate oxidative stress, and regulate intestinal flora; additionally, we summarize the mechanism in order to provide a reference for MEHH phenolic acids in the treatment of glycolipid metabolism diseases.

## 1. Introduction

**Figure 1:**
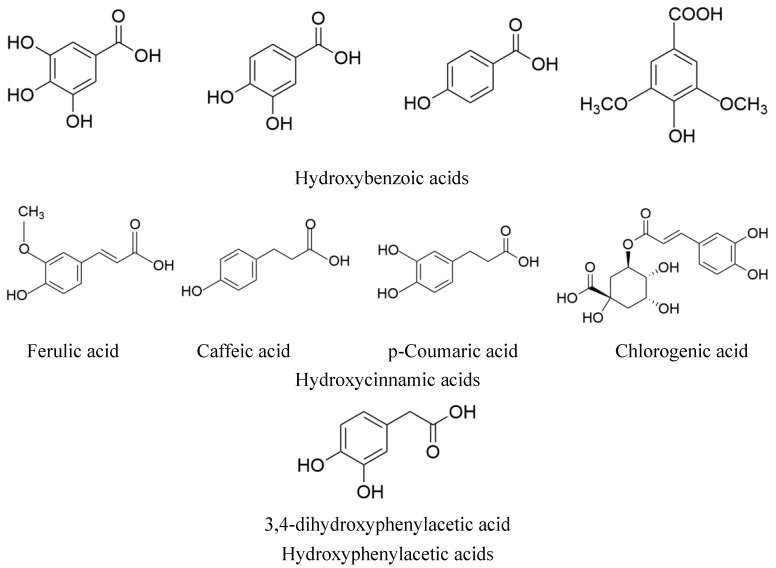
The structure of phenolic acid compounds in MEHHs.

Glycolipid metabolism disorder is a disorder of the glucose and fat metabolism in the body caused by a variety of factors, such as genetics and the environment, and its pathological mechanisms include oxidative stress, inflammation, insulin resistance, dyslipidemia, and intestinal flora imbalance. Clinical manifestations include high blood glucose, high blood lipids, non-alcoholic fatty liver disease, atherosclerosis, and other conditions [1]. These disorders are often interpreted in traditional Chinese medicine (TCM) as a dysfunction of the internal organs caused by factors like an uncontrolled diet, emotional impairment, and excessive work. Traditional Chinese medicine has advantages in treating glycolipid metabolism disorders, such as containing overall, multitarget, and multilevel effects. The main components of Chinese medicines used for the treatment of glycolipid metabolism disorders include phenolic acids, polysaccharides, flavonoids, alkaloids, and saponins. With changes in people’s lifestyle and dietary structure, the proportion of unhealthy individuals is increasing, and the traditional Chinese medicine health concept of “medicine comes from food, food has medicinal effects, and medicine has dietary habits” is becoming increasingly popular. Chinese medicine with the same origin as food refers to Chinese medicines that can be used for both the clinical treatment of diseases and daily consumption, including edible animals and plants, fungi, and seasoning spices, such as ginseng, ginger, chrysanthemum, hawthorn, Sichuan pepper, and *Angelica sinensis* [2]. The phenolic acids of medicinal and food plants are diverse and structurally complex. By reviewing the literature, we found that 70 species of medicinal plants with phenolic acid components have been reported, and the total number of phenolic acids was 167. There were 45 kinds of hydroxybenzoic acid, 113 kinds of hydroxycinnamic acid, 8 kinds of hydroxyphenyl acetic acid, and 1 kind of other phenolic acid [3]. The chemical structures of the phenolic acid compounds are shown in Figure 1.

Phenolic acids in medicinal and edible homologous plants exhibit a range of pharmacological activities, such as anti-inflammatory, antioxidant, antitumor, and anti-infective properties [4]. Consuming beverages, fruits, and vegetables that are rich in phenolic acids may decelerate the onset and progression of chronic ailments, including obesity, cardiovascular disease, diabetes, and cancer [5]. Research has shown that the phenolic acids in MEHHs may prevent and ameliorate disorders in glucose and lipid metabolism by modulating glucose and lipid metabolism, insulin signaling, reducing insulin resistance, curtailing inflammation and oxidative stress, and regulating gut flora [6,7,8,9,10,11].

In this review, we examine the potential role of MEHHs phenolic acids in the pathogenesis of glucose and lipid metabolism disorders. Our aim is to enrich the scientific understanding of MEHHs phenolic acids in ameliorating the homeostatic imbalance of glycolipid metabolism and to provide a theoretical foundation for future research.

## 2. Mechanism of Action of MEHHs Phenolic Acids in Regulating Glucose and Lipid Metabolism Disorders

A literature review reveals that phenolic acids in MEHHs are highly effective in treating diseases related to glucose and lipid metabolism, such as diabetes, atherosclerosis, hyperlipidemia, obesity, and non-alcoholic fatty liver disease. The primary mechanisms include regulating glucose and lipid metabolism, modulating insulin signaling, enhancing insulin sensitivity, reducing insulin resistance, inhibiting inflammatory responses, mitigating oxidative stress, and modulating gut microbiota; the specific mechanism is shown in Table 1.

### 2.1. Regulates Glucose Metabolism

Glucose metabolism, the primary metabolic pathway in the body, primarily functions to supply energy. Under normal conditions, glucose derived from ingested food is absorbed by the intestines and transported to the liver via the venous bloodstream. In the liver, some glucose is converted into liver glycogen for storage, while the remainder is released into the bloodstream to be utilized by other parts of the body. Additionally, other monosaccharides such as mannose, fructose, and galactose undergo conversion in the liver into glucose post-absorption from the intestines. Disorders in glucose metabolism, which include abnormalities in the structure, function, and concentration of hormones or enzymes that regulate the metabolism of glucose, fructose, and galactose, along with pathophysiological changes in tissues and organs, are prevalent in conditions such as diabetes, non-alcoholic fatty liver disease, and obesity [64]. Notably, phenolic acids such as chlorogenic acid (CGA), ferulic acid (FA), caffeic acid (CA), gallic acid (GA), rosmarinic acid (RA) and salvianolic acid B (SalB), etc., derived from MEHHs were found to modulate abnormal glucose metabolism.

#### 2.1.1. Inhibition of α-Amylase and α-Glucosidase Activity to Reduce Carbohydrate Cleavage

Starch is the most significant carbohydrate in the daily diet. The digestive enzymes α-amylase and α-glucosidase break starch into glucose, which is then absorbed through the small intestine, causing an increase in blood sugar levels. In the gut, α-glucosidase converts dietary carbohydrates into simple sugars for absorption [65]. Inhibiting the activity of α-amylase and α-glucosidase can reduce postprandial blood glucose levels, thereby helping to prevent and slow the onset and progression of diabetes mellitus [66]. Consequently, targeting α-glucosidase and α-amylase is a crucial strategy for controlling blood glucose [67]. Current medications such as acarbose, voglibose, and miglitol effectively inhibit α-amylase and α-glucosidase; however, their side effects restrict clinical use [68].

Research indicates that CA and CGA inhibit α-amylase and α-glucosidase, exerting antidiabetic effects by enhancing their capacity to scavenge DPPH free radicals in a dose-dependent manner [12]. Similarly, FA promotes hepatic glycogen synthesis, elevates glucokinase activity, and substantially reduces α-amylase and α-glucosidase activity [15,16]. Adisakwattana et al. identified CA and FA as the most potent inhibitors of α-glucosidase among eleven cinnamic acid derivatives, which contribute to their antidiabetic properties by inhibiting this enzyme [13]. Furthermore, caffeic acid significantly (*p* < 0.05) decreases α-amylase and lipase activities [14].

#### 2.1.2. Enhancing the Expression or Translocation of Glucose Transporters (GLUT) to Augment Glucose Uptake

The metabolism of glucose depends on cellular uptake, yet glucose cannot directly traverse the lipid bilayer of the cell membrane. Instead, glucose entry requires the facilitation of the glucose transporter (GLUT) present on the membrane. Enhancing the expression or activity of GLUT constitutes a primary mechanism by which cells modulate glucose uptake [14,69]. Of note, GLUT4 is an essential transporter that primarily facilitates glucose uptake in muscle and adipose tissue through insulin action. Conversely, GLUT2 primarily enhances glucose uptake in the liver and pancreatic β cells [70,71].

CA enhances the expression of GLUT4 protein in adipose tissue, restores AMPK phosphorylation, suppresses the expression of its direct substrate ACC, elevates CPT-1a expression, and reduces the activities of HMG-CoA reductase and SREBP-2 [17]. Moreover, CA improves the protein expression of IR and its tyrosyl phosphorylation, upregulates PI3K expression, activates downstream signaling molecules, increases GS expression, stimulates glycogen synthesis, enhances GLUT-2 expression, and boosts glucose uptake, thus mitigating insulin resistance [18]. Both CGA and CA increase glucose uptake in HepG2 cells by elevating GLUT4 expression and translocation [19]. RA ameliorates insulin resistance by reducing phosphoenolpyruvate carboxykinase (PEPCK) expression in the liver and augmenting GLUT4 expression in muscle [20].

#### 2.1.3. Inhibition of Gluconeogenesis, Promotion of Glycogen Synthesis, and Regulation of Blood Glucose Levels

Gluconeogenesis and glycogen metabolism are two critical metabolic pathways that collaboratively regulate blood sugar and energy balance. Gluconeogenesis involves converting non-carbohydrate substances into glucose, predominantly occurring in the liver through two main steps: initially, PEPCK transforms oxaloacetate into phosphoenolpyruvate; subsequently, phosphoenolpyruvate is converted into glucose-6-phosphate, and various free glucose molecules are regenerated [72]. Glycogen synthesis and degradation are controlled by intricate mechanisms that cater to the body’s energy demands and maintain stable blood glucose levels. Glycogen synthesis primarily takes place in the liver and muscle tissues, where phosphoglucomutase 1 (PGM1) converts G6P to G1P. This is followed by the addition of glucose residues, supplied by uridine diphosphate glucose pyrophosphate, to the glycogen chain by GYS1 [73]. Glycogenolysis is facilitated by enzymes such as glycogen phosphorylase and glycogen debranching enzyme, with glycogen phosphorylase playing a role in glycolysis by breaking down glycogen into G1P, which is then converted back to G6P by PGM1 [74,75]. The regulation of glycogen metabolism occurs at multiple levels, including gene expression, hormonal regulation, and enzymatic activity, which is controlled by phosphorylation/dephosphorylation and allosteric mechanisms. Glycogen synthase is believed to be activated by insulin, whereas glycogen phosphorylase is activated by glucagon [21]. Diabetes mellitus (DM) is marked by insulin resistance and impaired glucose metabolism, primarily in the liver, leading to reduced insulin sensitivity, diminished hepatic glycogen synthesis, and inhibited gluconeogenesis, which exacerbates glucose and lipid dysregulation.

**Figure 2:**
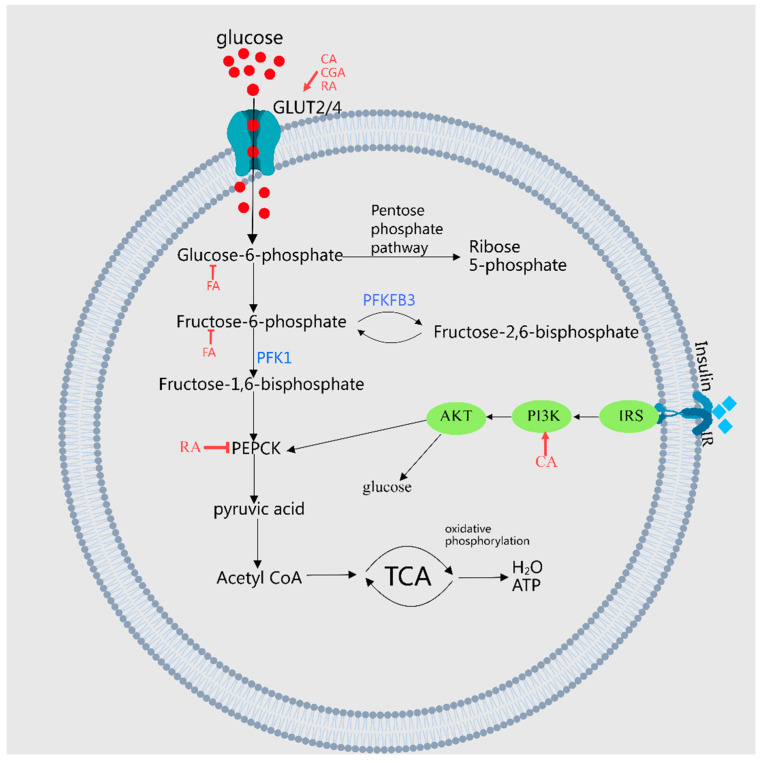
A demonstration of MEHHs’ phenolic acids regulating pathways in glucose metabolism. The arrow (→) indicates enhancement, while the barred line (┤) signifies inhibition. Glucose metabolism encompasses the synthesis, cleavage, transport, and storage of glucose in living organisms, involving critical pathways such as glycolysis, the tricarboxylic acid cycle, and the processes of glycogen synthesis and breakdown, along with gluconeogenesis. MEHHs’ phenolic acids facilitate glucose uptake, inhibit gluconeogenesis, and enhance glycogen synthesis by increasing the expression or translocation of glucose transporters GLUT4 and GLUT2, thereby balancing blood glucose levels.

FA enhances glucose utilization by inhibiting key enzymes such as glycogen phosphorylase, glucose-6-phosphatase, and fructose-1,6-bisphosphatase [16], thereby increasing glycogen synthesis and activating glucokinase [21]. Moreover, FA suppresses the protein expression of hepatic gluconeogenesis enzymes, including PEPCK and glucose-6-phosphatase (G6Pase) [22]. GA decreases the expression of proteins associated with hepatic gluconeogenesis like glyco-1,6-bisphosphatase in high-fat diet (HFD) rats, while enhancing the expression of hepatic glycogen synthase and glycolysis-related proteins, such as hexokinase, phosphofructokinase, and aldolase, thus mitigating hyperglycemia in rats [23]. SalBreduces the size of diabetic atherosclerotic plaques by lowering the expression of advanced glycation end products (AGEs) within the plaques [24]. Protocatechuic acid (PCA) ameliorates blood glucose levels in diabetic mice by reducing AGEs, glycosylated albumin, type IV collagen, and TGF-β1 in streptozotocin (STZ)-induced diabetic mice [25]. Lastly, the phenolic acids in MEHH influence the glucose metabolism pathway, as depicted in Figure 2.

### 2.2. Regulates Lipid Metabolism

Lipid metabolism encompasses the digestion and absorption of lipids in the small intestine, their entry into the bloodstream via the lymphatic system utilizing lipoprotein transport, transformation by the liver, and storage within adipose tissue for use by tissues as needed. This process is governed by genetic factors, neurohumoral influences, hormones, enzymes, and organs such as the liver. Abnormalities in these factors can lead to lipid metabolism disorders and pathophysiological alterations in related organs, presenting as elevated levels of plasma total cholesterol, triacylglycerol, low-density lipoprotein cholesterol, and apolipoprotein B. These are significant risk factors for conditions such as obesity, dyslipidemia, non-alcoholic fatty liver disease, and atherosclerosis. Research has indicated that phenolic acids such as CA, CGA, RA, FA, P-Ca, VA, and SalB from MEHHs may regulate abnormal lipid metabolism.

#### 2.2.1. AMPK, SREBP1, and ACC Signaling Pathways

AMPK is a crucial molecule in regulating cellular energy homeostasis. It senses changes in the intracellular AMP/ATP ratio, activates during declines in cellular energy, promotes fatty acid oxidation and autophagy, and inhibits gluconeogenesis, as well as lipid and protein synthesis. Sterol regulatory element-binding protein-1c (SREBP-1c) is a significant transcriptional regulator of lipid synthesis, primarily controlling the synthesis of fatty acids and cholesterol. It suppresses the expression of acetyl-CoA and FAS, playing a role in fat synthesis and triglyceride accumulation. ACC, a rate-limiting enzyme in the fatty acid synthesis pathway, is phosphorylated by AMPK, resulting in the inhibition of fatty acid synthesis.

Ferulic acid (FA) reduces the expression of hepatic adipogenic genes such as SREBP1c, FAS, and ACC, upregulates the CPT1a gene and PPARα protein [22], and significantly increases the levels of AMPKα protein and phosphorylated AMPKα. FA modulates lipid metabolism through the AMPKα/SREBP1/ACC1 signaling pathway [26]. Its role in lipid homeostasis is linked to a decreased expression of genes like SREBP1C, FAS, and ACC [27]. SalB may protect the liver by influencing the AMPK pathway, enhancing autophagy, reducing oxidative stress and inflammation, and mitigating liver damage in ApoE^−/−^ mice [28]. RA and its metabolites upregulate the proteins p-AMPK, p-SREBP-1c, and p-ACC, downregulate ACC and FAS proteins, and inhibit fatty acid synthesis both in vitro and in vivo. Rosemary extract (CA, RA) inhibits SREBP-1c-mediated adipogenesis by activating AMPK, thereby improving hepatic steatosis [29]. CA and CGA decrease hepatic FAS, HMG-CoA reductase, and ACAT activity [30], with CA also suppressing the expression of SREBP1, Fas, ACC, and SCD1 in the liver tissue of obese mice [31,32]. p-Coumaric acid (P-Ca) increases AMPK phosphorylation in a dose-dependent manner in differentiated L6 skeletal muscle cells, enhances ACC phosphorylation, and reduces glucose production by inhibiting gluconeogenesis [35]. CGA notably inhibits the activities of FAS, HMGCR, and ACAT, and boosts the expression of PPARα in the liver [33], also inhibiting HMGCoA activity and enhancing CPT activity by activating AMPK, thus promoting lipid metabolism, reducing TC synthesis, and attenuating fat absorption to combat obesity [34]. Vanillic acid (VA) suppresses ACC activity by promoting AMPK phosphorylation, thus inhibiting adipogenesis, reducing Malonyl-CoA production, and fostering lipid oxidation, which helps to alleviate hepatic steatosis [36].

CGA initially enhances the deacetylation activity of the SIRT1 protein by upregulating its expression. Subsequently, LKB1 is activated through phosphorylation, which, in turn, mediates the activation of AMPK via downstream phosphorylation. This activation leads to an increased expression of proteins MLYCD and CPT1α, facilitating the β-oxidation of fatty acids, while simultaneously reducing the expression of lipid synthesis proteins SREBP1c and FAS. As a result, lipid synthesis is inhibited and FFA-induced lipid deposition in HepG2 cells is attenuated through the SIRT1-LKB1-AMPK pathway [40]. PCA inhibits the phosphorylation of NF-κB in ApoE^−/−^ mice and enhances the expression of phosphorylated STAT6. Furthermore, PCA suppresses M1 polarization through the PI3K/Akt-NF-κB-SOCS1 pathway and mitigates atherosclerosis by promoting M2 polarization via the STAT6-PPARγ pathway [76]. SalB activates and upregulates SIRT1 and SIRT3; however, the effect on SIRT3 is diminished when the expression of SIRT1 is interfered with. The hepatoprotective effect of SalB is closely associated with the activation of the SIRT1/SIRT3 signaling pathway [77].

#### 2.2.2. Enhancing the Oxidative Activity of Beta Fatty Acids and Expression of PPARs

The β-oxidation of fatty acids is a crucial process in lipid metabolism, involving the breakdown of fatty acids into smaller units to generate energy. The regulation of this process is mediated by the peroxisome proliferator-activated receptor (PPAR), which includes three subtypes: PPAR-α, PPAR-β, and PPAR-γ. The activation of PPARA leads to increased peroxidase activity and the upregulation of genes such as carnitine palmitoyl transferase (CPT-1), leptin, and insulin receptors, thereby reducing lipogenesis and insulin resistance.

**Figure 3:**
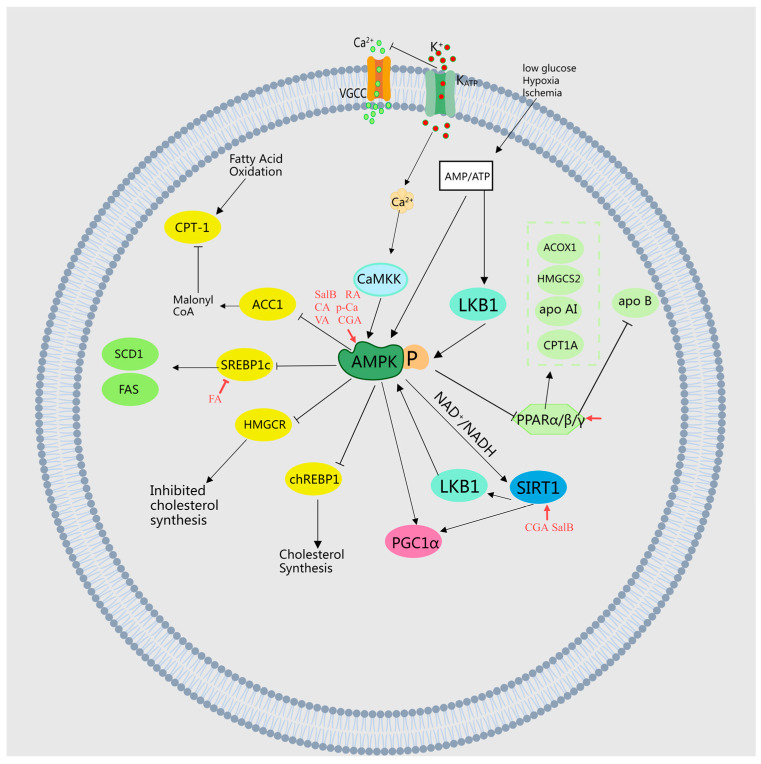
Illustration of MEHHs’ phenolic acids regulating lipid metabolism pathways. The arrow (→) indicates enhancement, while the barred line (┤) signifies inhibition. Lipid metabolism, a complex process, involves the synthesis, cleavage, transport, and storage of fats in living organisms. It includes the β-oxidation of fatty acids, cholesterol production, and lipoprotein metabolism, all finely regulated by hormones such as insulin and glucagon. These are crucial for maintaining energy balance, cellular structure, and function. The dysregulation of these pathways can lead to obesity, non-alcoholic fatty liver disease, and other metabolic disorders.

It has been discovered that FA competitively inhibits the activity of valonate-5-pyrophosphate dehydrogenase in the liver [15], enhances the activity of β-oxidation genes such as CPT1A, and increases the expression of PPARα in liver tissue [27], thereby inhibiting cholesterol synthesis and achieving lipid-lowering effects. Moreover, P-Ca elevates the expression of CPT-1 mRNA and PPARα in a dose-dependent manner, enhancing insulin sensitivity [35]. Similarly, CA and CGA amplify fatty acid β-oxidative activities and stimulate PPAR α expression in the liver, thus improving lipid metabolism in mice with obesity induced by a high-fat diet [30]. CGA also increases the expression levels of genes involved in fatty acid metabolism, including Cpt1a, Cpt1b, and Fgf21, while decreasing the expression levels of Pparγ1, Pparγ2, and their target genes Cd36, Fabp4, and Mgat1, thereby ameliorating hepatic steatosis and insulin resistance [38]. Protocatechic acid (PCA) exhibits antidiabetic effects through the inhibition of renal AR, SDH, GLI, PKC, PPAR-γ, and RAGE expressions [39]. RA significantly mitigates non-alcoholic fatty liver disease (NAFLD) by repairing mitochondrial damage and modulating the YAP1/TAZ-PPARγ/PGC-1α signaling pathway [40]. Danshensu increases the expression of LCAT and CYP7A1 genes and proteins in liver tissue, which upregulates apolipoprotein AI, downregulates apo B, promotes HDL-C transport, and reduces TC, TG, and LDL-C, thereby lowering lipid levels in hyperlipidemic rats [41]. Luo et al. reported that FA reduced hepatic steatosis and activated liver PPARα targets (CPT1A, ACOX1, and HMGCS2) in mice fed a high-fat diet, indicating that FA effectively prevents HFD-induced NAFLD by activating PPARα, increasing hepatic energy expenditure, and reducing intrahepatic TG accumulation [37]. MEHHs’ phenolic acids regulate lipid metabolism pathways, as illustrated in Figure 3.

### 2.3. Regulates Insulin Signaling, Improves Insulin Sensitivity and Improves Insulin Resistance

Insulin is the primary hormone that regulates essential energy functions, including glucose and lipid metabolism, and is the sole endogenous hormone in the body that reduces blood sugar levels. It activates the insulin receptor (IR) tyrosine kinase, leading to the aggregation and phosphorylation of various substrate docking proteins, such as the insulin receptor substrate (IRS) protein family. Notably, PI3K plays a crucial role in insulin function, primarily through the activation of Akt. This activation of Akt facilitates glycogen synthesis by inhibiting GSK-3 [78]. Additionally, insulin signaling promotes growth and mitosis, primarily mediated by the Akt cascade and the activation of the Ras/MAPK pathway. It enhances cell survival by activating the anti-apoptotic factor Bcl2 and inhibiting several pro-apoptotic factors, including Bax and Caspase-3. In the context of insulin resistance, impaired insulin signaling pathways lead to elevated blood glucose levels, disrupted glucose and lipid metabolism, and the exacerbated progress of diabetes [79].

**Figure 4:**
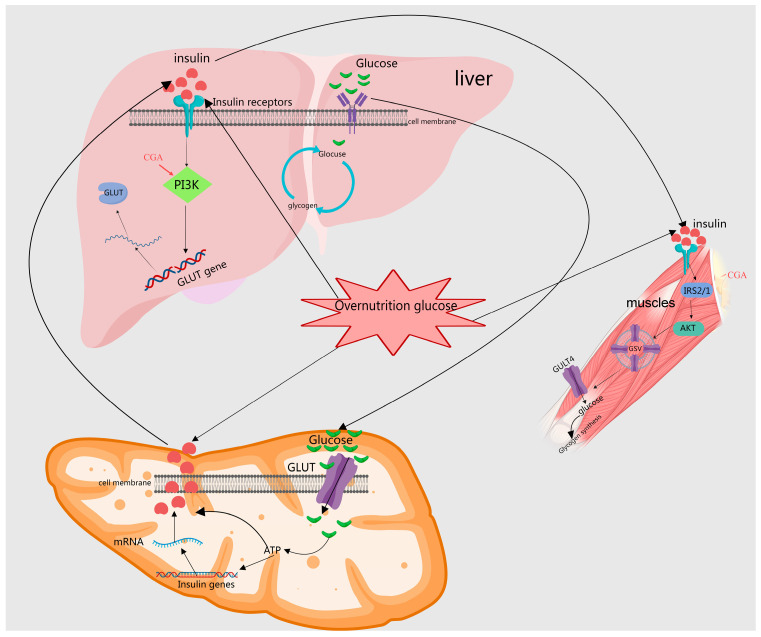
Depiction of how MEHHs phenolic acids regulate the insulin pathway. The arrow (→) indicates enhancement. The insulin signaling pathway plays a crucial role in regulating glucose and lipid metabolism within the body. MEHHs’ phenolic acids lower blood sugar levels by enhancing pancreatic β-cell secretion and promoting glucose uptake, glycogen synthesis, fatty acid synthesis, and storage. In the liver, muscle, and adipose tissue, these phenolic acids facilitate glucose utilization and glycogen synthesis through the activation of specific signal transduction pathways, such as the PI3K-Akt pathway, while concurrently inhibiting gluconeogenesis.

Studies have demonstrated that MEHHs’ phenolic acids, including CA, CGA, and SalB, enhance insulin sensitivity, modulate insulin signaling, and ameliorate insulin resistance. Specifically, CA significantly improves the functionality and morphology of pancreatic β cells in type II diabetic rats [14,42]. SalB effectively inhibits the activation of JNK and NF-κB in pancreatic tissues, decreases the expression of pro-apoptotic proteins Bax and Bim, increases the levels of the anti-apoptotic protein Bcl-2, and significantly reduces the activities of caspase-9 and caspase-3, which in turn reduces cell apoptosis, improves insulin resistance, and thus lowers blood glucose levels in rats [44,45,80]. Additionally, CGA suppresses autophagy by upregulating protein expression in the IR, IRS-1, PI3K, and Akt pathways, inhibiting the JNK pathway and improving insulin resistance [23]. MEHHs’ phenolic acids have been shown to regulate the glucose–insulin pathway, as illustrated in Figure 4.

### 2.4. Inhibits Inflammatory Responses

When metabolic disorders occur, adipose tissue releases various inflammatory mediators such as adipokines, which promote the recruitment and activation of inflammatory cells. Imbalances in glycolipid metabolism can lead to the accumulation of advanced glycation end products (AGEs) and free fatty acids, which directly bind to cell receptors and activate inflammatory signaling pathways. In a state of insulin resistance, while insulin signaling is impaired, insulin levels remain elevated; this hyperinsulinemia can promote the production and release of inflammatory factors. Dyslipidemia, characterized by hypertriglyceridemia and low levels of high-density lipoprotein cholesterol (HDL-C), can result in lipid deposition in non-adipose tissues, thereby activating an inflammatory response [81]. Inflammatory factors such as tumor necrosis factor-alpha (TNF-α), interleukin 6 (IL-6), and highly sensitive C-reactive protein (hs-CRP) play a significant role in the onset and progression of these diseases [82]. Traditional Chinese medicine (TCM), with its multiple targets and pathways, has been shown to ameliorate metabolic disorders by modulating inflammatory mediator levels. Notably, MEHHs phenolic acids—including CA, FA, RA, PCA, Danshensu, CGA, PMCA, SA, SalB, and other phenolic acids—exhibit inhibitory effects on inflammation.

#### 2.4.1. Accessing TLRs, NF-κB, and NLRP3 Pathways

Toll-like receptors (TLRs), prototypical pattern recognition receptors (PRRs), identify pathogen-associated molecular patterns (PAMPs) in microorganisms and damage-associated molecular patterns (DAMPs) in injured tissues. This recognition initiates the activation of intracellular signaling cascades, stimulates the immune response, and triggers the release of various inflammatory mediators [83]. Among the numerous TLR subtypes, TLR4 is particularly crucial in various inflammatory diseases [84] and its activation sets off the downstream NF-κB signaling pathway [85]. In most human cells, the NF-κB receptor remains inactive until stimulated, at which point NF-κB pairs with p65 to form a dimer that enters the nucleus to regulate the expression of target genes [86].

It has been demonstrated that CA reduces serum TNF-α and IL-6 levels, decreases serum LPS and hepatic TLR4 expression, and inhibits the upregulated expression of liver tissue SREBP1, Fas, ACC, and SCD1 induced by HFD. Additionally, it suppresses the activation of phosphorylated NF-κB p65 in liver tissues [46], thereby exerting a protective effect against HFD-induced NAFLD by curbing pro-inflammatory LPS release and lipid synthesis [31]. Danshensu modulates atherosclerosis in rats by downregulating cleaved caspase-3 protein expression, upregulating BCL-2 protein expression, reducing serum IL-6 and CRP levels, and suppressing the expression of TLR2, TLR4, p-IĸB, and NF-ĸB p65, with enhanced effects at higher doses [48]. Similarly, CGA reverses HFD-induced TLR4 signaling activation and reduces TNF-α and IL-6 expression [49]. Moreover, syringic acid (SA) significantly downregulates liver inflammation genes such as TLR4, MYD88, NF-κB, TNF-α, and IL6, and adipogenic genes including Cidea, Pparγ, Srebp-1c, Srebp-2, Fasn, and Hmgcr. It upregulates fatty acid oxidation genes like Pparα, Acsl, Cpt1, and Cpt2, thereby inhibiting liver inflammation and lipid synthesis while promoting fatty acid oxidation to address obesity induced by a high-fat diet in mice [52]. CA also notably reduces pro-inflammatory factors such as IL-6, TNF-α, and MCP-1, regulates cholesterol metabolism, diminishes lipid plaque accumulation, and slows atherosclerosis progression [47].

The antidiabetic properties of RA are attributable to its inhibition of NF-κB and MAPK expression [39]. In non-alcoholic fatty liver disease, SalB reduces the expression levels of NF-κB p65, IL-6, and TNF-α in the liver, mitigating liver damage [28]. PCA demonstrates anti-inflammatory effects by decreasing the release of IL-1β, TNF-α, and PGE2 in the brain and reducing NF-κB-binding activity, contributing to its anti-atherosclerotic actions [53]. PCA supplementation curtails IL-1β expression and NF-κB activation in lesions, inhibits the macrophage expression of IL-1b/IL-1β mRNA and protein, and initiates NF-κB activation through the upregulation of MERTK and MAPK 3/1, thereby lessening atherosclerosis severity [55]. In vitro, PCA reduces monocyte adhesion to aortic cells and NF-κB activation, inhibits the expression of aortic adhesion molecules, decreases VCAM-1 and ICAM-1 expression levels in the aorta, and prevents the development of early atherosclerotic lesions in ApoE-deficient mice [5,53]. FA lowers blood glucose and MDA levels in the liver, kidney, and serum, and reduces NF-κB expression in alloxan-induced diabetic mice [54].

The nucleotide-binding oligomerization domain-like receptor protein 3 (NLRP3) inflammasome, comprising NLRP3 receptors, CARD-containing apoptosis-associated speckle-like proteins (ASCs), and cysteine-containing aspartate proteolytic enzyme 1 (caspase-1), plays a critical role in mediating Aβ-induced microglial inflammation and is among the most extensively studied inflammasomes [87]. Salvianolic acid A (Sal A) mitigates the progression of atherosclerosis by suppressing NLRP3 inflammasome expression and reducing inflammation [56]. Furthermore, research on a high-fat diet-induced rat model of NAFLD revealed that Sal A significantly decreases the expression of TXNIP and inhibits the activation of the NLRP3 inflammasome and the nuclear translocation of ChREBP, indicating that Sal A’s protective effects on rat NAFLD are mediated through the modulation of the TXNIP-NLRP3 and TXNIP-ChREBP pathways [57].

#### 2.4.2. MAPK Signaling Pathway

Pro-apoptotic signaling molecules such as c-Jun N-terminal kinase (JNK) and p38 within the mitogen-activated protein kinase (MAPK) signaling pathway contribute to hyperglycemia-induced apoptosis and are linked to diabetes mellitus and its complications [88]. The phosphorylation of JNK facilitates its translocation from the cytoplasm to the nucleus, enhancing the phosphorylation of serine at positions 63 and 73 at the amino terminus of c-Jun. This increase amplifies the transcriptional activity of kinase protein 1 (AP-1) and regulates the gene expression of pro-inflammatory factors associated with JNK, such as IL-1 and TNF-α [89,90]. These events trigger more severe inflammatory responses and promote the apoptosis of pancreatic β cells.

**Figure 5:**
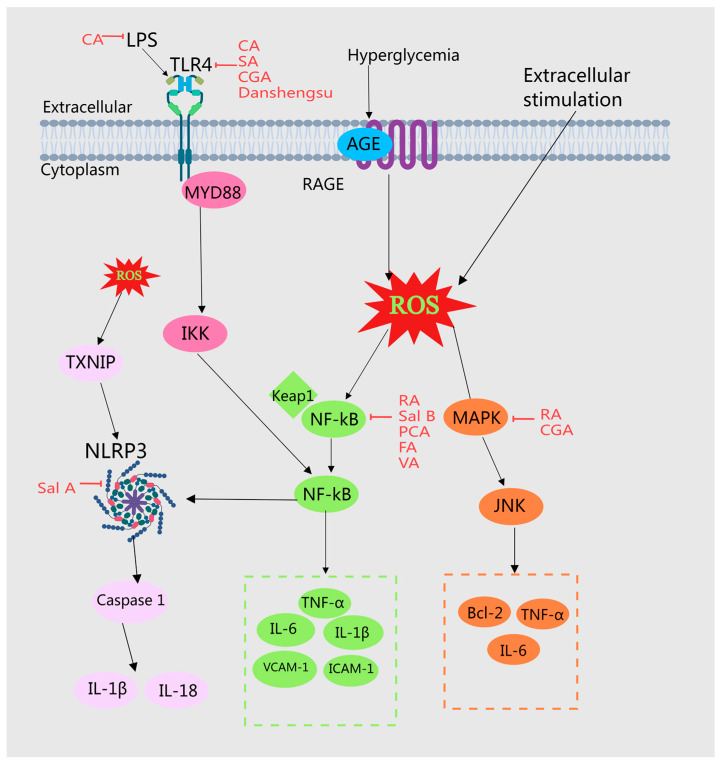
Illustration of MEHHs’ phenolic acids mitigating the inflammatory response by modulating various pathways. The arrow (→) indicates enhancement, while the barred line (┤) signifies inhibition. Disruptions in glycolipid metabolism can result in the accumulation of advanced glycation end products (AGEs) and free fatty acids, which may bind directly to cellular receptors and activate inflammatory signaling cascades. In conditions of insulin resistance, insulin signaling is compromised; however, insulin levels remain elevated, promoting hyperinsulinemia, which can further stimulate the production and release of inflammatory mediators. MEHHs’ phenolic acids counteract inflammatory responses and improve glucose and lipid metabolism disorders by inhibiting signaling pathways such as TLRs, NF-κB, NLRP3, and MAPK.

CGA treatment significantly inhibited the increase in P-JNK1/JNK1 levels induced by a high-fat diet. It also prevented the conversion of LC3-I to LC3-II and reduced the expression of Beclin-1, ATG3, and ATG5. Through the inactivation of the JNK pathway, CGA suppresses autophagy, consequently ameliorating liver injury and insulin resistance [50]. Furthermore, CGA dose-dependently decreased the expression levels of TNF-α, TNFR-1, Fasl, Fas, Caspase-8, Bax, Caspase-3, NF-kB, IL-6, and P53 in liver tissues, while it increased Bcl-2 expression, thereby alleviating NAFLD symptoms caused by a high-fat diet, reducing hepatocyte apoptosis, and providing a protective effect on hepatocytes [51]. MEHHs phenolic acids modulate different pathways to inhibit the inflammatory response, as depicted in Figure 5.

### 2.5. Inhibition of Oxidative Stress

Oxidative stress (OS) describes the physiological and pathological responses of cells and tissues induced by the production of reactive oxygen species (ROS) and reactive nitrogen radicals (RNS) due to harmful stimuli from both internal and external environments. These reactive species can directly or indirectly oxidize or damage DNA, proteins, and lipids, thereby inducing gene mutations, protein denaturation, and lipid peroxidation. Consequently, they are considered significant risk factors for diseases such as diabetes mellitus, as well as cardiovascular and cerebrovascular disorders [91]. Research has demonstrated that phenolic acids from MEHHs, including SalB, Sal A, FA, PMCA, GA, CA, Danshensu, and PCA, possess the capability to inhibit oxidative stress.

#### 2.5.1. The Nrf2 Signaling Pathway

Nuclear factor E2-related factor 2 (Nrf2) is a crucial transcription factor that typically binds to Keap1 in the cytoplasm and undergoes rapid degradation via the ubiquitin proteasome pathway. Under stimulation from reactive oxygen species (ROS) or other nucleophiles, Nrf2 dissociates from Keap1 and is activated through phosphorylation. Subsequently, it translocates to the nucleus where it competes with p65/p50 to activate the transcription factor CBP, concurrently inhibiting the binding of p65/p50 to target genes, and diminishing the transcription of TNF-a, IL-1b, and IL-6. This results in the suppression of the transcriptional and inflammatory responses mediated by p65/p50. Hence, the activation and nuclear translocation of Nrf2 are critical processes in the regulation of the Nrf2 pathway [92].

CGA ameliorates endothelial dysfunction in diabetic mice by increasing the expression of Nrf2 and its downstream target proteins HO-1, NQO1, and GPx1 in a dose-dependent manner, while also decreasing superoxide levels in HUVECs [58]. P-Ca reduces serum TC levels, enhances liver CAT and GSH-Px activities, and significantly upregulates the expression of Nrf2, SOD, HO-1, and NQO-1. These changes suggest that P-Ca may facilitate the recovery from hyperlipidemic steatohepatitis in mice by improving lipid peroxidation [59].

#### 2.5.2. Endoplasmic Reticulum Stress

The endoplasmic reticulum (ER) serves as the central regulator of the cell, overseeing protein folding and assembly, lipid metabolism, and calcium storage to maintain intracellular homeostasis. Impairment from conditions such as diabetes disrupt this balance, triggering an adaptive protective response known as endoplasmic reticulum stress (ERS) [93]. Short-term, mild ERS can be mitigated through the cell-associated degradation pathway (ERAD), whereas persistent, intense ERS initiates the unfolded protein response (UPR) pathway. The UPR functions as the sensor and protein regulator for the ER, tightly associated with immunoglobulin Bip in its resting state. The alterations in cellular glucose and lipid metabolism induced by diabetes impair ER protein synthesis, leading to an accumulation of numerous unfolded and misfolded proteins in the ER lumen, thus disrupting the ER’s environmental homeostasis and inducing ERS. Subsequently, the UPR dissociates from Bip and activates related signaling pathways, enhancing the cell’s capacity to clear misfolded and unfolded proteins and reducing ER stress [94,95]. Treatment with CA in AML12 hepatocytes significantly lowers the levels of ER stress markers such as BIP, ATF4, CHOP, GADD34, and XBP-1 protein [32]. Furthermore, CA mitigates the progression of diabetic atherosclerosis by diminishing ER stress and reducing glycosylated LDL-induced inflammatory stress in human endothelial cells [60]. CA also effectively manages ER stress both in vivo and in vitro by modulating the UPR pathways PERK, IRE1α, and ATF6α, thereby alleviating diabetic cardiomyopathy [61]. Additionally, insulinemia can induce ER stress, and VA alleviates this stress by modulating the expression of proteins associated with ER stress, including p-IRE1α, XBP1, p-PERK, p-eIF2α, and CHOP [96].

#### 2.5.3. Regulation of Oxidation-Related Factors

**Figure 6:**
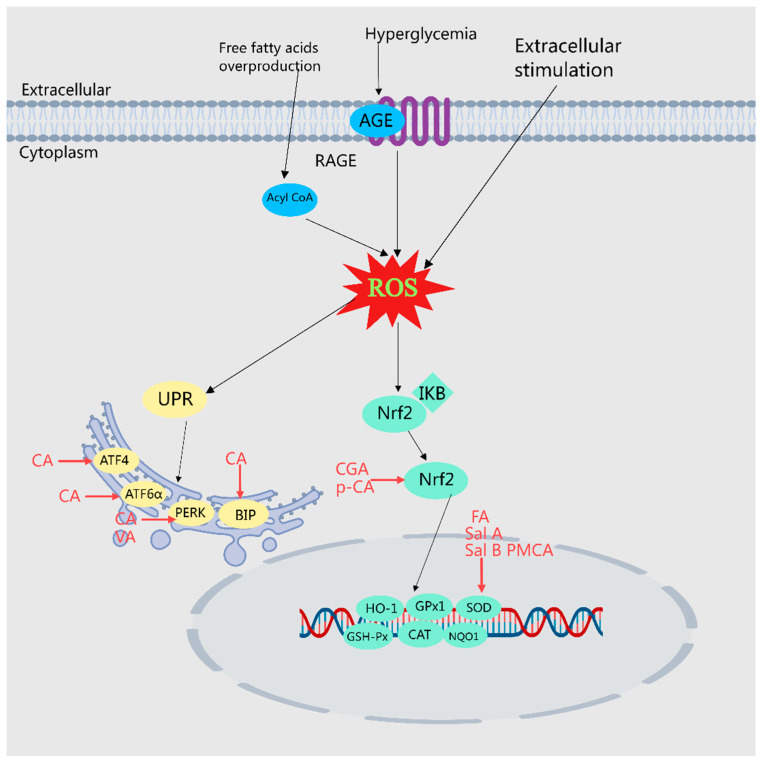
Illustration of MEHH phenolic acids mitigating oxidative stress by modulating various pathways. The arrow (→) indicates enhancement. Oxidative stress denotes the physiological and pathological responses of cells and tissues to the accumulation of reactive oxygen species (ROS) and reactive nitrogen radicals (RNS) stimulated by harmful internal and external environmental factors. MEHHs phenolic acids counteract oxidative stress and improve disorders in glucose and lipid metabolism by activating Nrf2, reducing ER stress, and regulating associated oxidative factors.

SalB significantly enhances aortic SOD activity and total antioxidant capacity, decreases NOX4 protein expression, and reduces MDA levels [45,97]. Sal A notably elevates SOD activity and lowers MDA levels in the DM model group, effectively mitigating oxygen free radical damage and enhancing resistance to lipid peroxidation [98]. FA plays a crucial role in atherosclerosis prevention by reducing plasma concentrations of TC and apo B and increasing liver activities of SOD, CAT, GSH-Px, and GR [99]. PMCA markedly decreases LDL-C and TG levels while increasing SOD, CAT, GSH, and TBARS, thus ameliorating hyperlipidemia [100]. GA administration in obese mice lowers TC and TG in serum and liver, elevates HDL-C, and increases liver activities of SOD and GSH-Px enzymes, along with a reduction in MDA levels [101]. CA reduces corticosterone, TNF-α, and IL-1α levels, significantly enhances serum insulin levels, and exerts antioxidant effects by lowering MDA and nitrite levels and increasing CAT and GSH levels [33]. Danshensu augments fatty acid β-oxidation and diminishes lipid droplet accumulation, improving NAFLD [102]. Treatment with CA or FA significantly reduces myocardial MDA, ROS, and GSSG levels, increases myocardial GSH levels, preserves GPX, SOD, and catalase activities, and downregulates mRNA expression of IL-1beta, IL-6, TNF-α, and MCP-1 in myocardial tissues of diabetic mice, thereby ameliorating diabetic cardiomyopathy [103]. CA markedly increases SOD, CAT, and GSH-Px activities in the liver and reduces lipid peroxidation levels [104]. CGA significantly restores HMG-CoA reductase activity in diabetic rats [105]. GA enhances glucose metabolism by boosting activities of the antioxidant-related proteins SOD, CAT, and GSH-Px [106]. CA elevates hepatic glycogen, SOD, and CAT activities in diabetic rats and reduces MDA levels, α-amylase, lipase, and AchE activities [14]. FA increases the activities of antioxidant enzymes such as GPX, SOD, and CAT and alleviates STZ-induced hepatic oxidative stress [27]. MEHH phenolic acids regulate oxidative stress by modulating different pathways, as illustrated in Figure 6.

### 2.6. Regulates Intestinal Flora

Recent studies have demonstrated that the microbial community within the gut, often referred to as the human “second genome”, influences the host’s glucose and lipid metabolism through various mechanisms, thereby regulating the onset and progression of associated diseases [107]. For instance, short-chain fatty acids (SCFAs), produced by the gut microbiota, serve as signaling molecules that impact glucose and lipid metabolism [71], insulin secretion, and the body’s energy equilibrium. Moreover, the diversity of gut microbiota in patients with obesity and diabetes is diminished, with specific microbiota linked to these disease states. Additionally, gut microbiota can affect cardiovascular health and insulin sensitivity by generating specific metabolites, such as trimethylamine N-oxide (TMAO) and imidazole propionic acid. It has been observed that phenolic acids, including CA, CGA, FA, and PCA, can modulate glucose and lipid metabolism by reshaping the intestinal microbiota [62]. This includes enhancing the composition and abundance of intestinal microbiota, rectifying the imbalance of intestinal bacteria, and repairing damage to the intestinal protective barrier.

**Figure 7:**
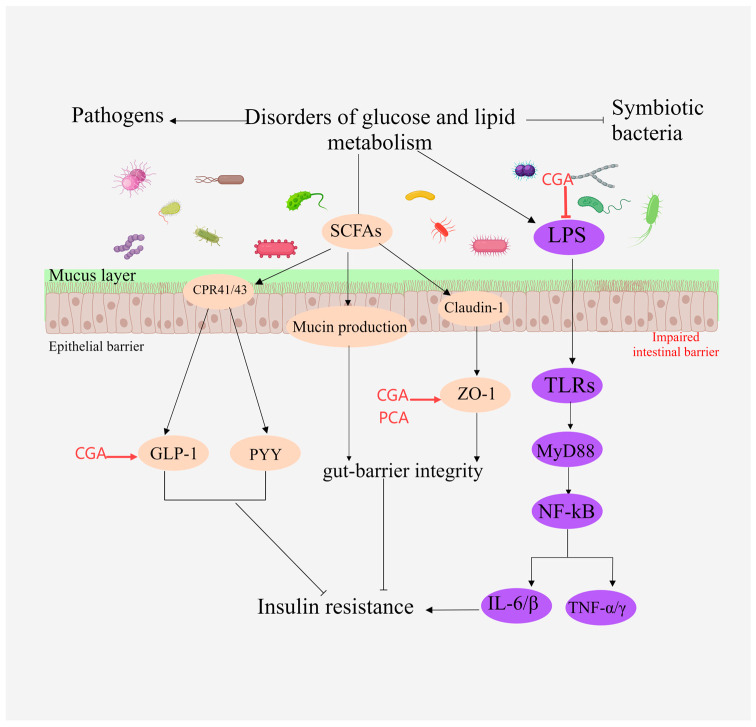
Illustration of the primary mechanisms through which MEHHs phenolic acids modulate intestinal microbiota. The arrow (→) indicates enhancement, while the barred line (┤) signifies inhibition. SCFAs activate GPR41 and GPR43 protein-coupled receptors in intestinal epithelial cells, inducing the production of YY peptide and GLP-1, and promoting the expression of intestinal tight junction proteins such as Zo-1 and occludin. By suppressing LPS expression, MEHHs phenolic acids increase the expression of ZO-1 and GLP-1, enhance the intestinal mucosal barrier, and mitigate disorders in glucose and lipid metabolism.

Caffeic acid (CA) can mitigate intestinal dysbiosis in high-fat diet (HFD) mice, notably enhancing ileal and colonic operational taxonomic units (OTUs), restoring microbial richness and diversity, and providing protection against HFD-induced NAFLD [31]. Chlorogenic acid (CGA) lowers *E. coli* levels by increasing bifidobacteria in feces; it also elevates the expression of tight junction proteins Occludin and ZO-1 in intestinal tissues, strengthens the intestinal mucosal barrier, decreases lipopolysaccharide (LPS) levels, and boosts glucagon-like peptide-1 (GLP-1), thereby ameliorating hepatic steatosis and inflammatory responses [49]. Ferulic acid (FA) triggers significant changes in the gut microbiota and fecal metabolites of atherosclerosis-susceptible mice, specifically reducing the relative abundance of *Bacteroides*, *Erysipelotrichaceae*, and *Ileobacteriaceae*, which correlate positively with blood lipid levels in these mice [26]. Protocatechuic acid (PCA) lowers the Firmicutes-to-Bacteroidetes ratio in the gut microbiota, increases the relative abundance of anti-inflammatory bacteria such as *Akkermansia* spp., and preserves the integrity of the ileal mucus layer. It also diminishes the infiltration of Gram-negative and Gram-positive bacteria in intestinal mucosal tissues, significantly boosts ZO-1 protein expression in ileal tissues, and reduces bacterial and endotoxin translocation to the liver, thus improving hepatic lipid metabolism [46]. CGA reverses gut microbiota dysbiosis induced by HFD, significantly inhibiting the growth of *Vibrio detrhioideae*, *Ruminococcaceae*, *Trichuraceae*, and *Erysipelotrichaceae*, and promoting *Lactobacillus* species, thereby alleviating HFD-induced obesity [63]. CGA uniquely alters the gut microbiota, which enhances HFD-induced obesity and associated glucose intolerance [108]. The primary mechanisms through which mono-esterified hydroxylated phenolic acids regulate the gut microbiota are depicted in Figure 7.

## 3. The Relationship between the Structure of MEHHs Phenolic Acids and the Regulation of Glucose and Lipid Metabolism Disorders

To a large extent, the biological activity of MEHHs is closely related to their structure, and structural modifications are often used in most studies to alter the structure of phenolic acids and enhance their biological activity. Phenolic acids are generally divided into two main groups: benzoic acids, containing seven carbon atoms (C6-C1), and cinnamic acids, consisting of nine carbon atoms (C6-C3) [109]. These natural compounds exist predominantly as hydroxybenzoic and hydroxycinnamic acids that may occur either in their free or conjugated forms. By comparing and summarizing the structure of MEHHs, the relationship between structure and biological activity can be inferred, which can provide a theoretical basis for further research on the regulation of glucose and lipid metabolism disorders by MEHHs.

### 3.1. Hydroxybenzoic Acids

Hydroxybenzoic acids are based on a hydroxybenzoic acid skeleton. The hydroxybenzoic acids can be divided into simple hydroxybenzoic acids, polyhydroxybenzoic acids, hydroxybenzoates, and hydroxybenzoate glycosides. According to reports, there are 18 types of simple hydroxybenzoic acids, 6 types of polyhydroxybenzoic acids, 12 types of hydroxybenzoates, and 9 types of hydroxybenzoate glycosides. Simple hydroxybenzoic acids are the most widely distributed (including vanillic acid, gallic acid, syringic acid, salicylic acid, protocatechuic acid, p-hydroxybenzoic acid, etc.) [3]. Studies have shown that the therapeutic effect of hydroxybenzoic acid compounds on diabetes is related to the number and location of hydroxyl groups [110].

#### 3.1.1. Number of Hydroxy Groups

The IC50 values of the phenolic acids with one or two hydroxyl groups on the benzene ring (4-hydroxybenzoic acid, 2,4-dihydroxybenzoic acid and 3,4-dihydroxybenzoic acid) were less than that of 3,4,5-trihydroxybenzoic acid, which has three hydroxyl groups. In addition, compared with 2,4-dihydroxybenzoic acid, the inhibition of 2,4,6-trihydroxybenzoic acid decreased when there was one more hydroxyl group. This is likely because the addition of hydroxyl groups reduces the hydrophobic properties of some phenolic acids, thus reducing their ability to pass through the entrance of the enzyme’s active site, which contains hydrophobic amino acid residues [111], making the inhibition of α-amylase less effective. However, the addition of hydroxyl groups enhanced the inhibitory activity of some compounds, such as 4-hydroxybenzoic acid, which had a significantly greater inhibitory capacity relative to benzoic acid. Additionally, 2,3,4-trihydroxybenzoic acid had an enhanced inhibitory capacity relative to 2,4-dihydroxybenzoic acid. For these structures, the hydroxyl groups may provide additional hydrogen binding opportunities to α-amylase, thus enhancing its activity of inhibiting α-amylase and playing an anti-diabetes role [112].

#### 3.1.2. Position of Hydroxyl Group

The inhibitory activity of hydroxybenzoic acid and its derivatives on alpha amylase seems to be related to the hydroxylation site. Adding a hydroxyl group at position 2 significantly increases the inhibition of alpha amylase. The 5-hydroxylation of the benzene ring greatly weakens the inhibitory ability of benzoic acid on alpha amylase. For example, 2,4-dihydroxybenzoic acid has a higher inhibitory effect on alpha amylase than 4-hydroxybenzoic acid, and 2,3,4-dihydroxybenzoic acid has a higher inhibitory effect on alpha amylase than 3,4-dihydroxybenzoic acid [113]. Removing the hydroxyl group at position 2 and adding a hydroxyl group at position 5 in the molecular structure of 2,3,4-trihydroxybenzoic acid significantly reduces its ability to inhibit alpha amylase. This indicates that the hydroxyl group at a specific position on the benzene ring is crucial for maintaining the inhibitory ability against alpha amylase [114].

### 3.2. Hydroxycinnamic Acids

Hydroxycinnamic acids are the most abundant and widely distributed phenolic acids. According to their structure, they can be divided into simple hydroxycinnamic acids, hydrogenated hydroxycinnamic acids, polyhydroxycinnamic acids, hydroxycinnamates, hydroxycinnamate glycosides, and hydroxycinnamate salts. Among the reported MEHP phenolic acids, there are 10 simple hydroxycinnamic acids, 7 hydrogenated hydroxycinnamic acids, 46 polyhydroxycinnamic acids, 26 hydroxycinnamates, 21 hydroxycinnamate glycosides, and 1 hydroxycinnamate salt. Among these, simple hydroxycinnamic acids and polyhydroxycinnamic acids are the most diverse. The most widely distributed simple hydroxycinnamic acids include caffeic acid, ferulic acid, and p-coumaric acid, which are distributed in 39, 31, and 28 MEHPs, respectively [3]. Most polyhydroxycinnamic acids have caffeic acid as the parent core, including caffeoylquinic acids which combine caffeic acid, quinic acid (chlorogenic acid), and rosmarinic acid the latter of which is a combination of caffeic acid and danshensu. The antioxidant capacity of hydroxycinnamic acid is related to the number and position of its hydroxyl groups.

#### 3.2.1. Number of Hydroxy Groups

The antioxidant activity of hydroxycinnamic acid seems to be largely influenced by the number of hydroxyl groups present on the aromatic ring [115]. Increasing the number of hydroxyl groups in hydroxycinnamic acid typically enhances its in vitro antioxidant capacity. Some studies have shown that trihydroxy groups (containing catechol moieties) have higher antioxidant activity than dihydroxy groups (containing catechol moieties) and monohydroxyphenolic acids [116]. This effect can be attributed to the formation of phenoxide radicals when hydroxycinnamic acid molecules are oxidized by ROS, which can be stabilized by adjacent electron-donating hydroxyl groups [117]. In addition, molecules with adjacent dihydroxy or 4-hydroxy-3-methoxy groups have higher antioxidant activity than molecules without these functions [118].

#### 3.2.2. Position of Hydroxyl Group

The phenolic hydroxyl group is the antioxidant active center of hydroxycinnamic acid derivatives, and derivatives with a catechol structure or electron-donating groups in the hydroxyl ortho position have higher antioxidant activity. The antioxidant activity of 4′-hydroxy-substituted derivatives is stronger than that of 3′-hydroxy-substituted derivatives. The conjugated structure of α, β-unsaturated acids on the benzene ring can enhance the antioxidant activity of phenolic compounds [119].

Phenolic compounds containing catechins have the function of chain breaking antioxidants [120]. Research has shown that caffeic acid exhibits the strongest antioxidant activity in various assays, such as lipid peroxidation and DPPH detection. Caffeic acid (dihydroxy substituted) has a stronger hypochlorous acid scavenging ability and singlet oxygen quenching ability than coumaric acid (monohydroxy substituted) [113]. The activity of hydroxycinnamic acid is highly dependent on the number of hydroxyl groups: compared to coumaric acid, adjacent dihydroxy derivatives (caffeic acid and chlorogenic acid) can serve as effective LDL peroxidation inhibitors [115]. Caffeic acid has ortho phenolic hydroxyl groups and exhibits strong antioxidant activity. Research has shown that the ortho or para substitution of phenolic hydroxyl groups can increase the antioxidant capacity of molecules, as it promotes the resonance dispersion of electrons, thereby more effectively stabilizing free radicals [121].

## 4. Summary and Outlook

As lifestyles and diets evolve, the incidence of sub-health and chronic diseases is rising annually. Consequently, the prevention of diseases and promotion of healthy living have become focal points of interest. The ancient Chinese concept of medicine and food homology posits that “food serves as medicine on an empty stomach, and for the sick, it is medicine”. In recent times, the health industry’s growth has spotlighted medicine and food homology products. These products hold significant value in fields such as medicine, food, and health care, offering vast potential for development and utilization. Since 2002, following the publication of the “List of Articles that Are Both Food and Medicine” by China’s former Ministry of Health, 110 Chinese herbal medicines have been cataloged, with plants making up nearly 95% of the list. Medicinal and edible homologous plants (MEHHs) are rich in active ingredients, including polyphenols, with phenolic acids being a major group. These compounds typically feature a carboxyl group and one or more hydroxyl groups attached to aromatic rings, and can be categorized into hydroxybenzoic acid, hydroxyphenylacetic acid, and hydroxycinnamic acid. They exhibit a range of biological activities such as antioxidant, antibacterial, antiviral, and anti-inflammatory properties. Given the complex role of phenolic acids in glycolipid metabolism, elucidating the mechanisms of MEHHs phenolic acids in treating diseases linked to glucose and lipid metabolism disorders is critical.

Firstly, the activity of MEHHs phenolic acids warrants a thorough investigation, which could be enhanced by employing modern omics technologies such as proteomics, metabolomics, transcriptomics, and genomics to accurately analyze their molecular mechanisms. Secondly, since the chemical structure influences biological activity, it is crucial to delve deeper into the relationship between the chemical structure and biological activity of MEHHs phenolic acids. Analyzing this structure–activity relationship (SAR) is vital for understanding the physicochemical properties and enhancing the quality control of MEHHs phenolic acids. The pathogenesis of glucose and lipid metabolism disorders is complex and involves multiple signaling pathways. MEHHs phenolic acids potentially offer a synergistic treatment for glycolipid metabolism diseases by simultaneously regulating glycolipid synthesis and insulin resistance through multiple pathways and targets. However, the factors influencing the synergistic effects of these pathways remain unclear, necessitating further research and experimentation. Additionally, while current studies on the regulation of glycolipid metabolism using MEHHs phenolic acids are predominantly conducted in animal models, there is a notable deficiency in clinical research, and some hypotheses remain speculative. Furthermore, the integration of research and the application of medicine and food homologous to Chinese medicine with societal needs is still limited.

Currently, several unexplored avenues remain in future research: (1) Although the continuous advancement of scientific and technological methods aids in exploring the unknown, there is still a scarcity of new technical test results that integrate known pathways and key targets; (2) it is crucial to utilize multidisciplinary approaches, such as integrating network pharmacology and other statistical analysis techniques, to delve deeply into the mechanisms of drug actions and further validate existing research findings; (3) present mechanistic studies primarily rely on animal model research, with a notable deficiency in studies based on clinical perspectives; (4) integrating new scientific research outcomes into clinical practice remains a vital future direction to enhance drug efficacy and bioavailability continuously. In summary, as technical methods advance, the pathological mechanisms and biological underpinnings of metabolic diseases will be more profoundly elucidated, offering significant references and insights for clinical practice. This paper reviews the mechanisms of MEHHs phenolic acids in treating glucose and lipid metabolism disorders, yet a more detailed explanation is still needed. Thus, further in-depth research on MEHHs phenolic acids is essential, particularly in identifying substances with significant activity, which will substantially contribute to their efficacy, metabolic pathways, pharmacological effects, and mechanisms of action. This research will be significantly beneficial for developing and utilizing MEHHs phenolic acids in treating diseases related to glucose and lipid metabolism disorders.

## Figures and Tables

**Table 1:** The main sources and mechanisms pathway of MEHHs.

No.	Components	MEHHs	Pathway	Mechanism Pathway	Reference
1	Caffeic acid	Cirsium setosum (Willd.) MB.; *Portulaca oleracea* L.; *Phyllanthus emblica* L.; *Citrus medica* L’	Regulates glucose metabolism	Inhibits α-amylase and α-glucosidase activity	[12,13,14]
2	Chlorogenic acid	*Cirsium setosum* (Willd.) MB.;	Regulates glucose metabolism	Inhibits α-amylase and α-glucosidase activity	[12]
3	Ferulic acid	*Dolichos lablab* L.; *Dimocarpus longan* Lour.; *Hippophae rhamnoides* L.	Regulates glucose metabolism	Inhibits α-amylase and α-glucosidase activity	[13,15,16]
4	Caffeic acid	*Lonicera japonica* Thunb; *Zingiber officinale* Rosc.; *Lycium barbarum* L.	Regulates glucose metabolism	Enhances the expression and translocation of GLUT4 and GLUT2 proteins	[17,18,19]
5	Chlorogenic acid	*Lycium barbarum* L.	Regulates glucose metabolism	Enhances the expression and translocation of GLUT4 proteins	[19]
6	Rosmarinic acid	*Perilla frutescens* (L.) Britt. (leaf)	Regulates glucose metabolism	Reduces PEPCK expression in the liver and increases GLUT4 expression in muscles	[20]
7	Ferulic acid	*Hippophae rhamnoides* L.; *Hovenia dulcis* Thunb.; *Morus alba* L. (leaf)	Regulates glucose metabolism	Inhibits glycogen phosphorylase, glucose-6-phosphatase, and fructose-1,6-diphosphatase activity Inhibits the protein expression of hepatic gluconeoxygenase, PEPCK, and G6Pase	[16,21,22]
8	Gallic acid	*Lycium barbarum* L.	Regulates glucose metabolism	Downregulates the expression of fructose-1,6-phosphatase, upregulate the expression of hexokinase, phosphofructokinase, and aldolase	[23]
9	Salvianolic acid B	*Angelica sinensis* (Oliv.) Diels.	Regulates glucose metabolism	Reduces the expression of AGEs within plaques	[24]
10	Protocatechuic acid	*Prunella vulgaris* L.	Regulates glucose metabolism	Reduces the levels of AGEs, glycosylated albumin and type IV collagen in STZ induced diabetes mice	[25]
11	Ferulic acid	Thunb.; *Morus alba* L. (leaf); *Curcuma Longa* L.; *Kaempferia galanga* L.	Regulates lipid metabolism	Regulates the AMPK α/SREBP1/ACC1 signaling pathway	[22,26,27]
12	Salvianolic acid B	*Mentha haplocalyx* Briq.	Regulates lipid metabolism	Regulates the AMPK pathway, enhances liver autophagy levels in ApoE^−/−^ mice, reduces liver oxidative stress and inflammation levels, and alleviates liver damage	[28]
13	Rosmarinic acid	*Prunella vulgaris* L.	Regulates lipid metabolism	Regulates the AMPK/SREBP1c signaling pathway	[29]
14	Caffeic acid	*Nelumbo nucifera* Gaertn. (leaf); *Chrysanthemum morifolium* Ramat.	Regulates lipid metabolism	Inhibits the expression of SREBP1, Fas, ACC, and SCD1 in the liver tissue of obese mice	[30,31,32]
15	Chlorogenic acid	*Cinnamomum cassia* Presl; *Zanthoxylum bungeanum* Maxim.	Regulates lipid metabolism	Activates AMPK-activated protein kinase, inhibits HMGCoA activity, enhances CPT activity	[30,33,34]
16	p-Coumaric acid	*Prunella vulgaris* L.	Regulates lipid metabolism	Dose-dependent increase in AMPK phosphorylation and ACC phosphorylation in differentiated L6 skeletal muscle cells	[35]
17	Vanillic acid	*Crataegus pinnatifida* Bge.	Regulates lipid metabolism	Activates AMPK phosphorylation and inhibits ACC activity	[36]
18	Ferulic acid	*Dolichos lablab* L.; *Kaempferia galanga* L.	Regulates lipid metabolism	Inhibits the activity of hydroxyglutaryl-5-pyrophosphate dehydrogenase in the liver, increases the activity of β-oxidation gene CPT1A and the expression of PPAR α in liver tissue	[15,27,37]
19	p-Coumaric acid	*Prunella vulgaris* L.	Regulates lipid metabolism	Enhances the expression of CPT-1 mRNA and PPAR α	[35]
20	Caffeic acid	*Angelica sinensis* (Oliv.) Diels.	Regulates lipid metabolism	Enhances the activity of fatty acid β oxidation and stimulates the expression of liver PPAR α	[30]
21	Chlorogenic acid	*Prunella vulgaris* L.; *Angelica sinensis* (Oliv.) Diels.	Regulates lipid metabolism	Enhances the activity of fatty acid β oxidation and stimulates the expression of liver PPAR α. Increases the expression level of genes involved in fatty acid metabolism	[30,38]
22	Protocatechuic acid	*Lycium barbarum* L.	Regulates lipid metabolism	Inhibits the expression of AR, SDH, GLI, PKC, PPAR-γ, and RAGE	[39]
23	Rosmarinic acid	*Vigna umbellata* Ohwi et Ohashi	Regulates lipid metabolism	Regulates the YAP1/TAZ-PPAR γ/PGC-1 α signaling pathway	[40]
24	Danshensu	*Mentha haplocalyx* Briq.; *Prunella vulgaris* L.	Regulates lipid metabolism	Increases the levels of LCAT and CYP7A1 genes and proteins in liver tissue, upregulates apolipoprotein apo AI and downregulates apo B	[41]
25	Caffeic acid	*Phyllanthus emblica* L.; *Mentha haplocalyx* Briq.	Regulates insulin signaling and improves insulin sensitivity	Improves the function and morphology of pancreatic β cells in type II diabetes rats	[14,42]
26	Chlorogenic acid	*Lycium barbarum* L.; *Prunella vulgaris* L.	Regulates insulin signaling and improves insulin sensitivity	Upregulates protein expression in the IR, IRS-1, PI3K, and Akt pathways, inhibits JNK pathway activation, and inhibits autophagy	[23,43]
27	Salvianolic acid B	*Angelica sinensis* (Oliv.) Diels.; *Mentha haplocalyx* Briq.	Regulates insulin signaling and improves insulin sensitivity	Inhibits the activation levels of JNK and NF-κ B in pancreatic tissue, downregulates the expression of pro-apoptotic proteins Bax and Bim, upregulates the level of anti-apoptotic protein Bcl-2, and significantly reduces the activity of Caspase-9 and Caspase-3	[44,45]
28	Caffeic acid	*Cirsium setosum* (Willd.) MB.; *Portulaca oleracea* L.; *Phyllanthus emblica* L.;	Inhibits inflammatory responses	Reduces the expression of TLR4 in the liver and inhibits the activation of phosphorylated NF-κ B p65 in liver tissue	[46,47]
29	Danshensu	*Mentha haplocalyx* Briq.; *Prunella vulgaris* L.	Inhibits inflammatory responses	Downregulates TLR2 and TLR4, p-I-κ B, and NF-κ B p65 protein expression	[48]
30	Chlorogenic acid	*Citrus medica* L.; *Prunus armeniaca* L.; *Hippophae rhamnoides* L.;	Inhibits inflammatory responses	Reverses the TLR4 signaling pathway induced by HFD Inhibits autophagy through JNK pathway inactivation	[49,50,51]
31	Salicylic acid	*Cichorium intybus* L.; *Hippophae rhamnoides* L.;	Inhibits inflammatory responses	Downregulates the expression of liver inflammation genes TLR4, MYD88, NF-κ B, and upregulates the expression of fatty acid oxidation genes Ppar α, Acsl, Cpt1, and Cpt2	[52]
32	Rosmarinic acid	*Perilla frutescens* (L.) Britt. (leaf)	Inhibits inflammatory responses	Inhibits NF-κ B and MAPK expression	[39]
33	Salvianolic acid B	*Angelica sinensis* (Oliv.) Diels.; *Mentha haplocalyx* Briq.	Inhibits inflammatory responses	Reduces the expression levels of NF-κ B p65, IL-6, and TNF-α in the liver	[28]
34	Protocatechuic acid	*Hippophae rhamnoides* L.; *Hordeum vulgare* L.; *Ziziphus jujuba* Mill.	Inhibits inflammatory responses	Reduces NF-κ B binding activity. By upregulating MERTK and MAPK 3/1, the activation of NF-κ B in macrophages is inhibited	[5,53,54]
35	Ferulic acid	*Laminaria japonica* Aresch.	Inhibits inflammatory responses	Significantly inhibits the expression of NF-κ B and significantly reduces the average concentration of MDA	[55]
36	Vanillic acid	*Crataegus pinnatifida* Bge.	Inhibits inflammatory responses	Downregulates NF-κ B and exerts anti-inflammatory effects	[36]
37	Salvianolic acid A	*Angelica sinensis* (Oliv.) Diels.	Inhibits inflammatory responses	Inhibits the expression of NLRP3 inflammasome and reduces inflammatory response Regulates the TXNIP-NLRP3 and TXNIP ChREBP pathways	[56,57]
38	Chlorogenic acid	*Lonicera japonica* Thunb.	Inhibition of oxidative stress	Increases the expression of Nrf2 and its downstream target proteins HO-1, NQO1, and GPx1	[58]
39	p-Coumaric acid	*Dimocarpus longan* Lour.	Inhibition of oxidative stress	Upregulates the expression of Nrf2, SOD, HO-1, and NQO-1	[59]
40	Caffeic acid	*Nelumbo nucifera* Gaertn. (leaf); *Chrysanthemum morifolium* Ramat.	Inhibition of oxidative stress	Significantly reduces the protein levels of ER stress markers BIP, ATF4, CHOP, GADD34, and XBP-1 Regulates the UPR pathways PERK, IRE1 α, and ATF6 α	[32,60,61]
41	Caffeic acid	*Nelumbo nucifera* Gaertn. (leaf); *Chrysanthemum morifolium* Ramat.	Regulates intestinal flora	Increases the ileum and colon OUT of HFD mice, restores the richness and diversity of the microbiota	[31]
42	Chlorogenic acid	*Cirsium setosum* (Willd.) MB.; *Crataegus pinnatifida* Bge.; *Portulaca oleracea* L.	Regulates intestinal flora	Increases the expression of tight junction proteins Occludin and ZO-1 in intestinal tissue, improves intestinal mucosal barrier, and reverses gut microbiota dysbiosis caused by HFD	[49,62,63]
43	Ferulic acid	*Kaempferia galanga* L.	Regulates intestinal flora	Specifically reduces the relative abundance of Bacteroides, Erysipella and Ileum, which are positively related to the blood lipid level of atherosclerosis mice	[26]
44	Protocatechuic acid	*Phyllanthus emblica* L.;	Regulates intestinal flora	Protects the integrity of the mucosal layer of the ileum, reduces the infiltration of Gram-negative and positive bacteria in the intestinal mucosal tissue, and significantly increases the expression of ZO-1 protein in the ileum tissue	[46]

## Data Availability

Data are contained within the article.

## Abbreviations

AS atherosclerosis	MAPK mitogen-activated protein kinase
Apo B apolipoprotein B	MDA malonaldehyde
AGEs advanced glycosylation end products	Nrf2 nuclear factor erythroid2-related factor 2
AMPK AMP-activated protein kinase	NAFLD non-alcoholic fatty liver disease
AchE acetylcholinesterase	NF-κB nuclear factor kappa-B
ACC acetyl CoA carboxylase	NLRP3 NOD-like receptor thermal protein domain associated protein 3
CAT catalase	OS oxidative stress
CA caffeic acid	PCA protocatechuic acid
CGA chlorogenic acid	P-Ca p-coumaric acid
CPT-1 carnitine palmitoyl transterase-1	PPARs peroxisome proliferators-activated receptors
DM diabetes mellitus	RA rosmarinus acid
FA ferulic acid	ROS reactive oxygen species
FFA non-esterified fatty acid	RNS reactive nitrogen species
GA gallic acid	SA salicylic acid
GSH-Px glutathione peroxidase	SOD super oxide dismutase
GSSG oxidized glutathione	STZ streptozotocin
GSH L-glutathione	SalA salvianolic acid A
GST glutathione S-transferase	SalB salvianolic acid B
HO-1 heme oxygenase-1	T1D type1 diabetes mellitus
HUVECs human umbilical vein endothelial cells	T2D type 2 diabetes mellitus
IL-1β interleukin-1β	TNF-α tumor necrosis factor alpha
IL eukin-6	TGF-β1 transforming growth factor-β
IR ischemia–reperfusion injury	TG triglyceride
ICAM-1 intercellular cell adhesion molecule-1	TC serum total cholesterol
LDL-C low density lipoprotein-cholesterol	VCAM-1 vascular cell adhesion molecule-1
